# 2-Cyano­anilinium nitrate

**DOI:** 10.1107/S1600536808023313

**Published:** 2008-07-31

**Authors:** Li-Jing Cui, Xiao-Chun Wen

**Affiliations:** a Ordered Matter Science Research Center, College of Chemistry and Chemical Engineering, Southeast University, Nanjing 210096, People’s Republic of China

## Abstract

In the title compound, C_7_H_7_N_2_^+^·NO_3_^−^, all atoms of the cation, with the exception of two H atoms of the NH_3_ group, lie on a mirror plane, while the anion lies across this plane with the N and one O atom on the mirror plane. In the crystal structure, the organic cations and NO_3_^−^ anions are linked by N—H⋯N and N—H⋯O hydrogen bonds, forming a two-dimensional network parallel to (100).

## Related literature

For the use of amino derivatives in coordination chemisty, see: Manzur *et al.* (2007 ▶); Ismayilov *et al.* (2007 ▶); Austria *et al.* (2007 ▶); Wen *et al.* (2008 ▶).
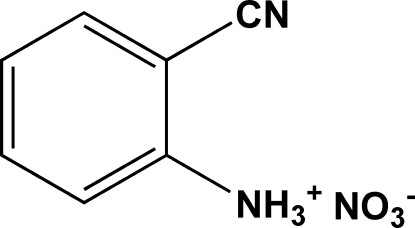


## Experimental

### 

#### Crystal data

C_7_H_7_N_2_^+^·NO_3_^−^
*M* *_r_* = 181.16
Orthorhombic, 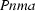

*a* = 16.373 (3) Å
*b* = 6.5627 (13) Å
*c* = 7.9948 (16) Å
*V* = 859.0 (3) Å^3^
*Z* = 4
Mo *K*α radiation
μ = 0.11 mm^−1^
*T* = 298 (2) K
0.25 × 0.25 × 0.15 mm

#### Data collection

Rigaku Mercury2 diffractometer
Absorption correction: multi-scan (*CrystalClear*, Rigaku, 2005 ▶) *T*_min_ = 0.927, *T*_max_ = 0.983
8381 measured reflections
1072 independent reflections
797 reflections with *I* > 2σ(*I*)
*R*_int_ = 0.041

#### Refinement

*R*[*F* ^2^ > 2σ(*F* ^2^)] = 0.063
*wR*(*F* ^2^) = 0.167
*S* = 1.12
1072 reflections
83 parameters
H atoms treated by a mixture of independent and constrained refinement
Δρ_max_ = 0.17 e Å^−3^
Δρ_min_ = −0.18 e Å^−3^

### 

Data collection: *CrystalClear* (Rigaku, 2005 ▶); cell refinement: *CrystalClear*; data reduction: *CrystalClear*; program(s) used to solve structure: *SHELXS97* (Sheldrick, 2008 ▶); program(s) used to refine structure: *SHELXL97* (Sheldrick, 2008 ▶); molecular graphics: *SHELXTL* (Sheldrick, 2008 ▶); software used to prepare material for publication: *SHELXTL*.

## Supplementary Material

Crystal structure: contains datablocks I, global. DOI: 10.1107/S1600536808023313/ci2636sup1.cif

Structure factors: contains datablocks I. DOI: 10.1107/S1600536808023313/ci2636Isup2.hkl

Additional supplementary materials:  crystallographic information; 3D view; checkCIF report

## Figures and Tables

**Table 1:** Hydrogen-bond geometry (Å, °)

*D*—H⋯*A*	*D*—H	H⋯*A*	*D*⋯*A*	*D*—H⋯*A*
N1—H1*A*⋯O1^i^	0.95 (3)	1.86 (3)	2.805 (2)	177 (2)
N1—H1*A*⋯N3^i^	0.95 (3)	2.56 (3)	3.4523 (13)	156 (2)
N1—H1*A*⋯O2^i^	0.95 (3)	2.61 (3)	3.2898 (7)	129 (2)
N1—H1*B*⋯O1^ii^	0.94 (5)	2.13 (4)	2.979 (3)	150 (1)
N1—H1*B*⋯O1^iii^	0.94 (5)	2.13 (4)	2.979 (3)	150 (1)
N1—H1*B*⋯N3^ii^	0.94 (5)	2.49 (5)	3.427 (4)	180 (3)

Symmetry codes: (i) 
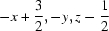
; (ii) 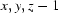; (iii) 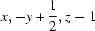.

**Fig. 1.**
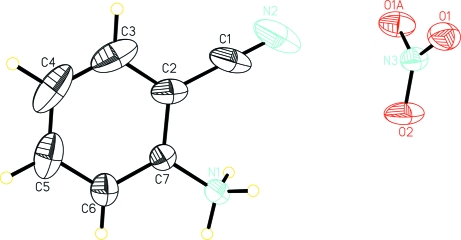
A view of the title compound with the atomic numbering scheme. Displacement ellipsoids are drawn at the 30% probability level.

**Fig. 2.**
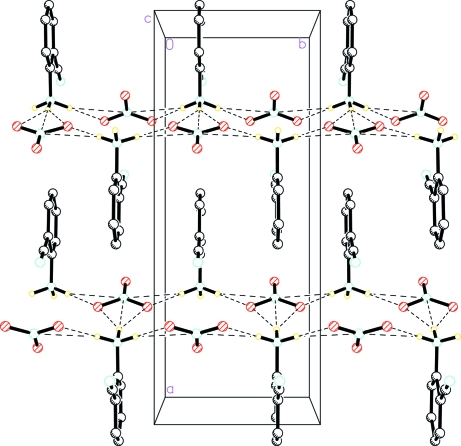
Part of the crystal packing of the title compound viewed along the c axis. H atoms not involved in hydrogen bonding (dashed lines) have been omitted for clarity.

## supplementary crystallographic information

### Comment

In the past five years, we have focused on the chemistry of amine derivatives because of their multiple coordination modes as ligands to metal ions and for the construction of novel metal-organic frameworks (Manzur *et al.* 2007; Ismayilov *et al.* 2007; Austria *et al.* 2007; Wen 2008). We report here the crystal structure of the title compound, 2-cyanobenzenaminium nitrate.

In the title compound (Fig.1), N atom of the amine group is protonated. The nitrile group is essentially coplanar with the benzene ring. Bond lengths and angles lie within normal ranges.

In the crystal structure, the organic cation and NO_3_^-^ anions are linked to form a two-dimensional network parallel to the (1 0 0) by N—H···N and N—H···O hydrogen bonds (Table 1, Fig.2).

### Experimental

2-Cyanobenzenaminium nitrate (3 mmol) was dissolved in ethanol (20 ml). The solution was allowed to evaporate to obtain colourless block-shaped crystals of the title compound.

### Refinement

C-bound H atoms were fixed geometrically (C-H = 0.93 Å) and treated as riding with *U*_iso_(H) = 1.2U_eq_(C). N-bound H atoms were located in a difference Fourier map and refined freely.

### Crystal data

**Table:**

C_7_H_7_N_2_^+^·NO_3_^–^	*F*_000_ = 376
*M**_r_* = 181.16	*D*_x_ = 1.401 Mg m^−^^3^
Orthorhombic, *P**n**m**a*	Mo *K*α radiation λ = 0.71073 Å
Hall symbol: -P 2ac 2n	Cell parameters from 1592 reflections
*a* = 16.373 (3) Å	θ = 2.5–27.4º
*b* = 6.5627 (13) Å	µ = 0.11 mm^−^^1^
*c* = 7.9948 (16) Å	*T* = 298 (2) K
*V* = 859.0 (3) Å^3^	Block, colourless
*Z* = 4	0.25 × 0.25 × 0.15 mm

### Data collection

**Table:**

Rigaku Mercury2 diffractometer	1072 independent reflections
Radiation source: fine-focus sealed tube	797 reflections with *I* > 2σ(*I*)
Monochromator: graphite	*R*_int_ = 0.041
Detector resolution: 13.6612 pixels mm^-1^	θ_max_ = 27.5º
*T* = 298(2) K	θ_min_ = 2.5º
ω scans	*h* = −20→21
Absorption correction: multi-scan(CrystalClear, Rigaku, 2005)	*k* = −8→8
*T*_min_ = 0.927, *T*_max_ = 0.983	*l* = −10→10
8381 measured reflections	

### Refinement

**Table:**

Refinement on *F*^2^	Secondary atom site location: difference Fourier map
Least-squares matrix: full	Hydrogen site location: inferred from neighbouring sites
*R*[*F*^2^ > 2σ(*F*^2^)] = 0.063	H atoms treated by a mixture of independent and constrained refinement
*wR*(*F*^2^) = 0.167	*w* = 1/[σ^2^(*F*_o_^2^) + (0.0725*P*)^2^ + 0.211*P*] where *P* = (*F*_o_^2^ + 2*F*_c_^2^)/3
*S* = 1.12	(Δ/σ)_max_ = 0.001
1072 reflections	Δρ_max_ = 0.17 e Å^−^^3^
83 parameters	Δρ_min_ = −0.18 e Å^−^^3^
Primary atom site location: structure-invariant direct methods	Extinction correction: none

### Special details

**Table:**

Geometry. All esds (except the esd in the dihedral angle between two l.s. planes) are estimated using the full covariance matrix. The cell esds are taken into account individually in the estimation of esds in distances, angles and torsion angles; correlations between esds in cell parameters are only used when they are defined by crystal symmetry. An approximate (isotropic) treatment of cell esds is used for estimating esds involving l.s. planes.
Refinement. Refinement of F^2^ against ALL reflections. The weighted R-factor wR and goodness of fit S are based on F^2^, conventional R-factors R are based on F, with F set to zero for negative F^2^. The threshold expression of F^2^ > 2sigma(F^2^) is used only for calculating R-factors(gt) etc. and is not relevant to the choice of reflections for refinement. R-factors based on F^2^ are statistically about twice as large as those based on F, and R- factors based on ALL data will be even larger.

### Fractional atomic coordinates and isotropic or equivalent isotropic displacement parameters (Å^2^)

**Table:**

	*x*	*y*	*z*	*U*_iso_*/*U*_eq_	
O1	0.76566 (11)	0.0885 (3)	0.9282 (2)	0.0793 (6)	
O2	0.82708 (18)	0.2500	0.7321 (3)	0.0951 (9)	
N3	0.78679 (15)	0.2500	0.8601 (3)	0.0603 (7)	
N1	0.68710 (15)	0.2500	0.2370 (3)	0.0571 (7)	
N2	0.6141 (4)	0.2500	0.6492 (5)	0.1409 (19)	
C1	0.5859 (3)	0.2500	0.5202 (5)	0.0962 (14)	
C2	0.5501 (2)	0.2500	0.3564 (4)	0.0714 (9)	
C3	0.4664 (3)	0.2500	0.3345 (9)	0.1171 (18)	
H3	0.4319	0.2500	0.4269	0.141*	
C4	0.4342 (2)	0.2500	0.1752 (11)	0.125 (2)	
H4	0.3779	0.2500	0.1609	0.150*	
C5	0.4835 (3)	0.2500	0.0394 (7)	0.0980 (14)	
H5	0.4609	0.2500	−0.0673	0.118*	
C6	0.5668 (2)	0.2500	0.0584 (4)	0.0690 (9)	
H6	0.6008	0.2500	−0.0347	0.083*	
C7	0.59927 (17)	0.2500	0.2164 (3)	0.0516 (7)	
H1A	0.7034 (16)	0.132 (5)	0.298 (3)	0.083 (8)*	
H1B	0.714 (3)	0.2500	0.134 (6)	0.104 (14)*	

### Atomic displacement parameters (Å^2^)

**Table:**

	*U*^11^	*U*^22^	*U*^33^	*U*^12^	*U*^13^	*U*^23^
O1	0.0975 (13)	0.0642 (11)	0.0763 (11)	0.0153 (9)	0.0278 (9)	0.0156 (8)
O2	0.0991 (19)	0.097 (2)	0.0893 (17)	0.000	0.0552 (15)	0.000
N3	0.0525 (14)	0.0693 (17)	0.0592 (14)	0.000	0.0098 (12)	0.000
N1	0.0533 (15)	0.0752 (18)	0.0426 (13)	0.000	0.0006 (11)	0.000
N2	0.205 (5)	0.161 (4)	0.057 (2)	0.000	0.034 (3)	0.000
C1	0.126 (4)	0.105 (3)	0.057 (2)	0.000	0.039 (2)	0.000
C2	0.074 (2)	0.068 (2)	0.072 (2)	0.000	0.0283 (18)	0.000
C3	0.067 (3)	0.125 (4)	0.159 (5)	0.000	0.047 (3)	0.000
C4	0.045 (2)	0.113 (4)	0.219 (7)	0.000	−0.008 (3)	0.000
C5	0.072 (3)	0.085 (3)	0.137 (4)	0.000	−0.040 (3)	0.000
C6	0.064 (2)	0.074 (2)	0.069 (2)	0.000	−0.0116 (16)	0.000
C7	0.0504 (15)	0.0517 (15)	0.0527 (16)	0.000	0.0010 (12)	0.000

### Geometric parameters (Å, °)

**Table:**

O1—N3	1.241 (2)	C2—C7	1.378 (4)
O2—N3	1.218 (3)	C3—C4	1.378 (8)
N3—O1^i^	1.241 (2)	C3—H3	0.93
N1—C7	1.448 (4)	C4—C5	1.353 (8)
N1—H1A	0.95 (3)	C4—H4	0.93
N1—H1B	0.94 (5)	C5—C6	1.371 (5)
N2—C1	1.130 (6)	C5—H5	0.93
C1—C2	1.434 (6)	C6—C7	1.371 (4)
C2—C3	1.382 (6)	C6—H6	0.93
			
O2—N3—O1^i^	121.32 (12)	C5—C4—C3	120.9 (4)
O2—N3—O1	121.32 (12)	C5—C4—H4	119.5
O1^i^—N3—O1	117.4 (2)	C3—C4—H4	119.5
C7—N1—H1A	109.7 (16)	C4—C5—C6	120.2 (5)
C7—N1—H1B	112 (3)	C4—C5—H5	119.9
H1A—N1—H1B	109 (2)	C6—C5—H5	119.9
N2—C1—C2	179.9 (5)	C7—C6—C5	119.2 (4)
C3—C2—C7	118.5 (4)	C7—C6—H6	120.4
C3—C2—C1	121.4 (4)	C5—C6—H6	120.4
C7—C2—C1	120.2 (3)	C6—C7—C2	121.4 (3)
C2—C3—C4	119.7 (4)	C6—C7—N1	119.4 (3)
C2—C3—H3	120.1	C2—C7—N1	119.2 (3)
C4—C3—H3	120.1		
			
C7—C2—C3—C4	0.0	C5—C6—C7—N1	180.0
C1—C2—C3—C4	180.0	C3—C2—C7—C6	0.0
C2—C3—C4—C5	0.0	C1—C2—C7—C6	180.0
C3—C4—C5—C6	0.0	C3—C2—C7—N1	180.0
C4—C5—C6—C7	0.0	C1—C2—C7—N1	0.0
C5—C6—C7—C2	0.0		

Symmetry codes: (i) *x*, −*y*+1/2, *z*.

### Hydrogen-bond geometry (Å, °)

**Table:**

*D*—H···*A*	*D*—H	H···*A*	*D*···*A*	*D*—H···*A*
N1—H1A···O1^ii^	0.95 (3)	1.86 (3)	2.805 (2)	177 (2)
N1—H1A···N3^ii^	0.95 (3)	2.56 (3)	3.4523 (13)	156 (2)
N1—H1A···O2^ii^	0.95 (3)	2.61 (3)	3.2898 (7)	129 (2)
N1—H1B···O1^iii^	0.94 (5)	2.13 (4)	2.979 (3)	150 (1)
N1—H1B···O1^iv^	0.94 (5)	2.13 (4)	2.979 (3)	150 (1)
N1—H1B···N3^iii^	0.94 (5)	2.49 (5)	3.427 (4)	180 (3)

Symmetry codes: (ii) −*x*+3/2, −*y*, *z*−1/2; (iii) *x*, *y*, *z*−1; (iv) *x*, −*y*+1/2, *z*−1.
